# Re-visiting the functional Relevance of the highly conserved Serine 40 Residue within HIV-1 p6^Gag^

**DOI:** 10.1186/s12977-014-0114-8

**Published:** 2014-12-19

**Authors:** Benjamin Radestock, Robin Burk, Barbara Müller, Hans-Georg Kräusslich

**Affiliations:** Department of Infectious Diseases, Virology, University Hospital Heidelberg, Im Neuenheimer Feld 324, D-69120 Heidelberg, Germany

**Keywords:** Human immunodeficiency virus, Gag, p6, Phosphorylation, Release, Serine 40

## Abstract

**Background:**

HIV-1 formation is driven by the viral structural polyprotein Gag, which assembles at the plasma membrane into a hexagonal lattice. The C-terminal p6^Gag^ domain harbors short peptide motifs, called late domains, which recruit the cellular endosomal sorting complex required for transport and promote HIV-1 abscission from the plasma membrane. Similar to late domain containing proteins of other viruses, HIV-1 p6 is phosphorylated at multiple residues, including a highly conserved serine at position 40. Previously published studies showed that an S40F exchange in p6^Gag^ severely affected virus infectivity, while we had reported that mutation of all phosphorylatable residues in p6^Gag^ had only minor effects.

**Findings:**

We introduced mutations into p6^Gag^ without affecting the overlapping *pol* reading frame by using an HIV-1 derivative where *gag* and *pol* are genetically uncoupled. HIV-1 derivatives with a conservative S40N or a non-conservative S40F exchange were produced. The S40F substitution severely affected virus maturation and infectivity as reported before, while the S40N exchange caused no functional defects and the variant was fully infectious in T-cell lines and primary T-cells.

**Conclusions:**

An HIV-1 variant carrying a conservative S40N exchange in p6^Gag^ is fully functional in tissue culture demonstrating that neither S40 nor its phosphorylation are required for HIV-1 release and maturation. The phenotype of the S40F mutation appears to be caused by the bulky hydrophobic residue introduced into a flexible region.

## Findings

HIV-1 assembly is driven by the viral Gag polyprotein. Gag is necessary and sufficient for particle formation, and is composed of four functional subunits. The N-terminal MA domain targets Gag to the plasma membrane, where ~2,500 Gag molecules form the curved, hexagonal immature lattice of HIV-1, which is stabilized by intermolecular CA domain interactions. The NC domain is responsible for packaging viral genomic RNA [1]. Crucial motifs for particle release lie within the 52 amino acids long C-terminal p6^Gag^ domain. This domain harbors so-called late domains, which recruit the cellular endosomal sorting complex required for transport (ESCRT) promoting abscission of progeny particles from the host cell. Other motifs in p6^Gag^ mediate incorporation of the viral accessory protein Vpr into HIV-1 particles [2-4]. During maturation, immature HIV-1 gains infectivity following proteolytic cleavage of Gag into its functional domains by the viral protease [1].

p6^Gag^ has been shown to be the predominant phosphoprotein in HIV-1 particles [5]. It is phosphorylated at several positions, including the highly conserved residue S40 [5-8]. S40 phosphorylation has been detected in infected cells and viral particles [7], and this residue can be phosphorylated by atypical protein kinase C (aPKC) *in vitro* [6].

To determine the role of p6 phosphorylation for HIV-1 replication, we had recently performed a comprehensive mutational analysis of p6 [7]. The use of an HIV-1_NL4-3_ based proviral plasmid with genetically uncoupled *gag* and *pol* open reading frames (ORFs) (pNL4-3_unc_) allowed us to freely introduce mutations in the p6^*gag*^ encoding region without affecting the *pol* ORF. In this context, we changed all phosphorylatable residues (i.e., Ser, Thr, Tyr) within p6^Gag^ with exception of the essential threonine in the PTAP late domain motif. The resulting virus, NL4-3_unc_FL exhibited no significant difference in replication capacity compared to wild-type. This result led us to conclude that p6 phosphorylation is dispensable for viral morphogenesis and replication in cell culture.

In contrast, previous studies had reported that an S40F change in p6^Gag^ impaired proteolytic maturation of Gag, reduced viral infectivity and delayed replication in T-cell lines [9,10]. Furthermore, enhanced membrane binding affinity of a synthetic p6 C-terminal fragment was observed *in vitro* upon substitution of Ser40 by Phe or upon adding a phosphate group to this residue [11]. The S40F exchange was furthermore shown to result in an enhanced interaction of Gag with the ESCRT-associated protein Alix [9]. Taken together, these studies suggested an important role of S40 in Gag assembly [9], viral maturation [10], Vpr incorporation [6], and p6 membrane binding [11], in apparent contradiction to our observation that an HIV-1 derivative carrying mutations at 12 positions within p6, including S40, was fully functional in cell culture [7].

A major difference between our work [7] and the studies reported by others [6,9-11] was that the latter employed a chemically drastic Ser to Phe exchange in order to maintain the amino acid sequence of the overlapping *pol* ORF, whereas the *gag-pol* uncoupling strategy allowed us to select the most conservative substitution, Ser to Asn. In order to resolve the apparent discrepancies between our study and data published by others, we performed a direct side-by-side comparison of viruses carrying either an Asn or a Phe residue at position 40 of p6^Gag^.

**Figure 1:**
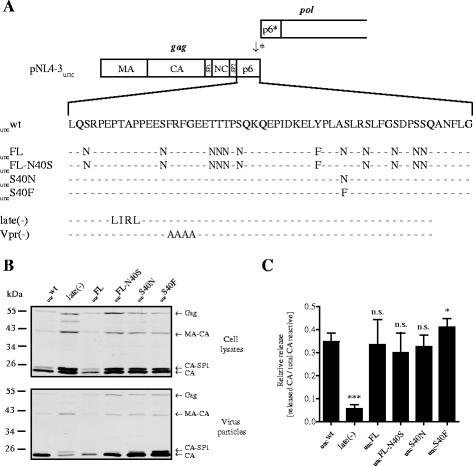
**HIV-1 p6** ^**Gag**^ **variants and their effect on Gag processing and viral release. (A)** Scheme of the *gag* and *pol* ORFs in the HIV-1_NL4-3unc_ genome [15]. The arrow with an asterisk indicates the frameshift signal at the 3’ end of *gag*. Amino acid sequences of the altered p6 sequences of the NL4-3_unc_ variants are shown below; mutated residues are indicated. The NL4-3 late(-) and Vpr(-) variants were constructed in the wild-type pNL4-3 proviral plasmid with overlapping *gag* and *pol* reading frames. **(B)** Gag processing and particle release efficiency. Virus particles were prepared by ultracentrifugation from the culture media of HEK 293 T cells transfected with the indicated proviral plasmids. Cell lysates (top panel) and virus particles (bottom panel) were analyzed for Gag-derived products by quantitative immunoblot (LiCor) using antiserum against HIV-1 CA. Positions of molecular mass standards are shown to the left, Gag-derived processing products are indicated to the right. **(C)** Quantitative immunoblotting of cell lysates and particles as shown in (B) was used to calculate the amount of released CA-containing proteins by quantification of band intensities using the LiCor Odyssey 3.0 software. Relative release was calculated by dividing the sum of CA-reactive band intensities in the particle fraction by total CA-reactive band intensities in cell lysates and particles. Mean values and SD from three independent experiments performed in duplicates are shown. *p*-values were calculated using an unpaired student’s *t*-test (n.s. > 0.05, * < 0.05, *** < 0.001).

The analysis included the previously described proviral plasmids pNL4-3_unc_ with uncoupled wild-type *gag* and *pol* and pNL4-3_unc_FL, in which all phosphorylatable residues in p6^Gag^ except for T8, which is required for L-domain function [12], had been changed to chemically similar, but not phosphorylatable residues [7]. A derivative of pNL4-3_unc_FL in which the substitution at position 40 of p6^Gag^ was reversed to the wild-type Ser-codon while retaining all other substitutions (pNL4-3_unc_FL-N40S) was also included (Figure 1A). Mutant viruses were produced by transfection of HEK 293 T cells [13] using calcium phosphate and tested for efficiency of particle formation, Gag processing, Vpr incorporation, and infectivity. Controls included a release deficient late domain-defective variant (NL4-3 late(-), [14]), a derivative carrying alanine substitutions in the FRFG motif of p6^Gag^ and impaired in Vpr incorporation (NL4-3 Vpr(-), [3]), and a derivative which does not express Vpr (NL4-3 ΔVpr).

For assessment of virus release, culture media were harvested 30 h post transfection (p.t.), cleared by brief centrifugation followed by ultracentrifugation through a 20% (w/w) sucrose cushion to pellet virus particles. Samples of cell and particle lysates were separated by SDS-PAGE and proteins were transferred to a PVDF membrane. HIV-1 CA-containing proteins were detected by quantitative immunoblotting using polyclonal sheep antiserum against recombinant CA (Figure 1B), and HIV-1 particle release was quantified by determining the ratio of the amount of pelletable extracellular CA-containing proteins over the total amount of CA-containing proteins (Figure 1C). As expected, NL4-3 late(-) showed strongly reduced particle release compared to wild-type, accompanied by a characteristic increase in the proportion of processing intermediates, in particular CA-SP1. Variants NL4-3_unc_FL, FL-N40S, and S40N displayed wild-type Gag processing and particle release. Increased amounts of the CA-SP1 processing intermediate were observed in case of the S40F variant, consistent with previous reports [9,10], and particle release was also slightly higher in this case (Figure 1B,C).

**Figure 2:**
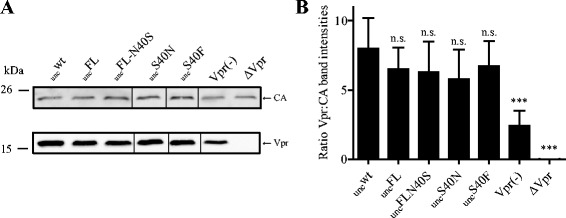
**Vpr incorporation into HIV-1 particles. (A)** Immunoblot analysis of Vpr incorporation. Virions were collected from culture media of HEK 293 T cells transfected with the respective proviral plasmids by ultracentrifugation through a sucrose cushion. NL4-3 Vpr(−) (generated by transferring a SpeI – AgeI fragment from pNL43_E-_R-_Luc3/p6:M1A ([3]) into pNL4-3) and NL4-3 ΔVpr were used as negative controls. Samples were separated by SDS-PAGE, and viral particles were analyzed by quantitative immunoblot (LiCor) using antisera against recombinant HIV-1 CA (top) and Vpr (bottom). The positions of molecular mass standards are shown on the left and proteins are indicated on the right. **(B)** Quantification of Vpr incorporation. The ratio of Vpr to CA represents relative signal intensities from quantitative immunoblots, as shown in panel A. Mean values and SD from three independent experiments performed in duplicates are shown. *p*-values were calculated using an unpaired student’s *t*-test (n.s. > 0.05, *** < 0.001).

Kudoh and coworkers reported that (i) an S40A substitution in p6^Gag^ abolished incorporation of exogenously expressed Vpr into virus-like Gag particles (VLPs) and (ii) a PKC inhibitor, presumed to prevent phosphorylation of S40, impaired HIV-1 replication in primary macrophages [6]. These authors proposed that S40 phosphorylation may be required for Vpr incorporation into HIV-1 particles, and may thus be functionally relevant for replication in non-dividing cells, where the accessory protein Vpr is required [16]. In our previous analyses, we had not observed a block of Vpr incorporation in the case of pNL4-3_unc_FL, where all phosphorylatable residues including S40 had been mutated [7]. This was confirmed in the present study, using a polyclonal rabbit antiserum raised against synthetic HIV-1 Vpr. Our analysis further revealed that neither the S40F nor the S40N exchange altered Vpr incorporation into HIV-1 (Figure 2). We conclude that the Vpr incorporation block reported before [6] is either a specific property of the S40A variant, or, more likely, represents a feature of the Gag/Vpr overexpression system used in the prior study that is not observed in the native viral context.

**Figure 3:**
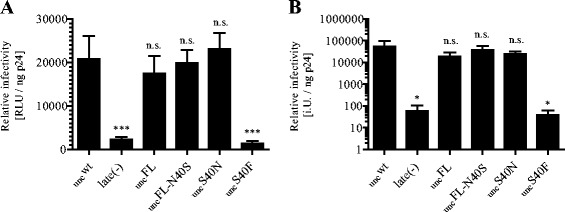
**Effect of p6 S40 substitutions on viral infectivity.** Viral particles were harvested from cell culture media of HEK 293 T cells transfected with proviral plasmids and tested for infectivity. Single round infectivity **(A)** was assessed by titration of viral particles on TZM-bl cells as described in the main text. Relative infectious titers on C8166 T-cells **(B)** were determined by endpoint titration. Values were normalized to p24 amounts determined by ELISA. Graphs show mean values and SD of one experiment using viral particles from six independent preparations. *p*-values in **(A)** and **(B)** were calculated using an unpaired student’s *t*-test (n.s. > 0.05, * < 0.05, *** < 0.001).

Single round infectivity of wild-type and mutant virus preparations was assessed by titration on HeLa TZM-bl indicator cells [17] (Figure 3A). Viral titers were determined by endpoint titration on C8166 T-cells [18] (Figure 3B). Neither single-round infectivity nor viral titer of the variants HIV-1_NL4-3unc_FL, FL-N40S, or S40N, respectively, differed significantly from that of wild-type HIV-1_NL4-3unc_ (Figure 3A,B). In line with published results [9,10], infectivity was severely decreased for the S40F variant with a similar reduction as observed for the release deficient late(-) control.

**Figure 4:**
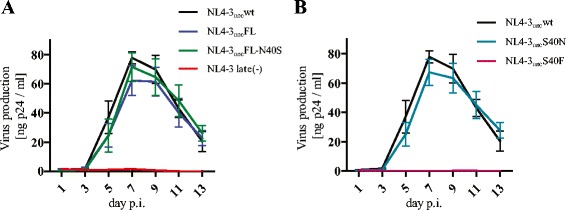
**Replication kinetics of HIV-1 variants in PBMC.** PBMC were isolated using buffy coats from healthy blood donors and stimulated for three days using PHA and IL-2. Subsequently, cells from three individual donors were mixed, and virus harvested from cell culture media of HEK 293 T cells transfected with the respective proviral plasmids was added in triplicates (0.1 ng p24 / 3 × 10^5^cells). Samples of cell culture media were collected at the indicated time points and virus production was measured by p24 ELISA. Results from one infection experiment using six independent virus preparations are shown; data represent mean values and SD. **(A)** Replication kinetics of HIV-1_NL4-3unc_wt, FL, FL-N40S, and NL4-3 late(-). **(B)** Replication kinetics of HIV-1_NL4-3unc_wt, S40N, and S40F.

Finally, we investigated the replication kinetics of the mutant HIV-1 panel in primary human peripheral blood mononuclear cells (PBMC) (Figure 4). PBMC obtained from three healthy blood donors were mixed, activated for 3 days with 2 μg/ml PHA (Sigma) and 10 μg/ml IL-2 (Biomol), and infected with virus obtained from transfected HEK 293 T cells. Virus replication was monitored over two weeks by collecting culture supernatant every second day and quantifying p24 release by ELISA. Replication of the late(-) (Figure 4A) and S40F variants (Figure 4B) was completely abolished in PBMC, consistent with their severe replication defect in established cell lines. The replication defect of the S40F mutant in PBMCs was even more severe than in a previous report analyzing replication in primary human lymphocyte aggregate cultures, where residual and delayed replication had been observed [10]. Most importantly, however, replication kinetics of the S40N, FL, and FL-N40S variants in PBMC were indistinguishable from wild-type HIV-1_NL4-3unc_ (Figure 4A, B), demonstrating that the serine residue itself and phosphorylation at this site are both dispensable for HIV-1 replication in primary T-cells.

In summary, our analyses confirm previous reports that an S40F substitution in p6^Gag^ of HIV-1 impairs Gag processing at the CA-SP1 site and severely affects or abolishes HIV-1 replication in cell lines and primary cells [9,10]. This effect is not due to either a requirement for the conserved serine residue at this position or for phosphorylation of S40. The conservative substitution of S40 by a chemically similar, but not phosphorylatable Asn residue had no effect on Gag processing or viral infectivity in cell lines or primary T-cells compared to wild-type HIV-1. Thus, the previously described replication defect of the S40F variant appears to be due to the replacement of the small, hydrophilic serine by a bulky hydrophobic phenylalanine residue, rather than indicating a requirement for S40. We did not analyze Gag membrane-binding properties, which had been reported to be affected by the S40F substitution or by introducing a phosphomimick in this position [11]. However, wild-type release, polyprotein processing and infectivity of all variants studied here except for S40F suggest that neither S40 nor its phosphorylation is needed for fully functional membrane binding of Gag. Furthermore, we did not observe a block in Vpr incorporation upon mutation of S40, which had been reported for an S40A variant in a previous study and had been proposed to result in impaired replication in primary macrophages [6]. While we cannot exclude that S40 in p6^Gag^ and/or its phosphorylation may be relevant in a different cell context (e.g., macrophages), we conclude that the phenotypes reported in previously published studies [6,9-11] were most likely caused by the specific mutation introduced and do not reflect the functional importance of Ser40 or its phosphorylation.
